# Prospective analysis on the gut microbiome and the risk of autoimmune rheumatic diseases in the population-based FINRISK 2002 cohort

**DOI:** 10.1093/rheumatology/keag371

**Published:** 2026-07-16

**Authors:** Hassan Diab, Li-Fang Yeo, Veikko Salomaa, Aki Havulinna, Leo Lahti, Katariina Pärnänen, Rob Knight, Joonatan Palmu, Teemu Niiranen

**Affiliations:** Department of Internal Medicine, University of Turku, Turku, Finland; Department of Internal Medicine, University of Turku, Turku, Finland; Department of Internal Medicine, University of Turku, Turku, Finland; Department of Public Health, Finnish Institute for Health and Welfare, Helsinki, Finland; Faculty of Medicine, Research Programs Unit, Clinical and Molecular Metabolism (CAMM), University of Helsinki, Helsinki, Finland; Department of Computing, University of Turku, Turku, Finland; Department of Computing, University of Turku, Turku, Finland; Department of Computing, University of Turku, Turku, Finland; Department of Microbiology, University of Helsinki, Helsinki, Finland; Department of Pediatrics, University of California San Diego, San Diego, CA, USA; Center for Microbiome Innovation, Joan and Irwin Jacobs School of Engineering, University of California San Diego, La Jolla, CA, USA; Department of Bioengineering, University of California San Diego, La Jolla, CA, USA; Department of Computer Science and Engineering, University of California San Diego, La Jolla, CA, USA; Halıcıoğlu Data Science Institute, University of California San Diego, La Jolla, CA, USA; Hong Kong University of Science and Technology Jockey Club Institute for Advanced Study, Hong Kong University of Science and Technology, Hong Kong SAR, China; Department of Internal Medicine, University of Turku, Turku, Finland; Department of Public Health, Finnish Institute for Health and Welfare, Helsinki, Finland; Heart Center, Turku University Hospital, Turku, Finland; Department of Internal Medicine, University of Turku, Turku, Finland; Department of Public Health, Finnish Institute for Health and Welfare, Helsinki, Finland; Division of Medicine, Turku University Hospital, Turku, Finland

**Keywords:** autoimmune rheumatic disease, gut microbiome, prospective study

## Abstract

**Objectives:**

To examine the long-term relationship between the gut microbiome and the risk of incident autoimmune rheumatic diseases (ARDs) in the general adult population.

**Methods:**

Participants of the FINRISK cohort (*N* = 6242) donated faecal samples in 2002 and were followed for incident ARD which was a composite outcome, defined as developing RA, AS or systemic connective tissue disorder. We used multivariable-adjusted models to assess the association of incident ARD with alpha diversity, community composition, prevalent taxa and prevalent predicted pathways.

**Results:**

Incident ARD was observed in 264 (4.2%) participants over a median follow-up of 19.8 years. The top species detected in the multivariable-adjusted models were *Scatocola faecipullorum*, *Sutterella wadsworthensis_A_*565807, *Alistipes_A_871404 indistinctus* and *CAG-217 sp000436335*. However, none of the associations reached statistical significance after FDR correction. Moreover, we did not find evidence of a statistically significant association between incident ARD and alpha diversity, community composition or prevalent predicted pathways in the age- and sex-adjusted or the multivariable-adjusted models.

**Conclusion:**

No evidence of association between baseline gut microbiome composition and the risk of incident ARDs (composite outcome) in the Finnish general adult population was detected in the current study. Our null findings, however, should be interpreted with caution since our study was limited by the use of a composite outcome (rather than using individual ARDs) and a single baseline measurement of the gut microbiome. More research efforts are still required to understand the prospective relationship between gut microbiome and individual ARDs.

Rheumatology key messages

- Here, no evidence on a prospective link between ARD and gut microbiome was detected.
- Low incidence of ARDs highlights the need for more research to understand ARD–gut microbiome relationship.

## Introduction

Extensive research has established a primary role of the human gut microbiome in promoting a healthy and balanced immune system [1, 2]. Alterations in the composition of the gut microbiome have been repeatedly linked to different inflammatory and autoimmune diseases such as autoimmune rheumatic disease (ARDs) [3–5]. For example, an increased abundance of *Prevotella* spp. has been observed in individuals with preclinical stage of RA and in patients with AS [4, 6]. Moreover, SLE patients have a less diverse gut microbiome and a different gut microbiome composition compared with healthy controls [5].

Numerous studies have investigated the link between the gut microbiome and ARDs [3–17]. The majority of these studies have been either cross-sectional and case-control studies or experimental studies that involve animal models. However, despite previous research efforts, the role of the gut microbiome as a risk factor for incident ARD in the general adult population remains poorly understood. Cross-sectional and case-control studies have linked changes in the gut microbiome composition to ARDs, but they cannot provide information about whether gut dysbiosis preceded or followed ARD development. Despite being observational, prospective cohort studies offer stronger evidence and better support for causal inferences and are less biased due to the prospective evaluation of the exposure.

Here, we investigated the association of gut microbiome diversity and composition with the risk of incident ARD (which was a composite outcome defined as developing at least one of the following diseases: RA, AS or other systemic connective tissue disorder [SCTD]) in 6242 participants of the FINRISK 2002 prospective population-based cohort.

## Methods

A detailed description of the methods is available in Supplementary Data S1. Briefly, the FINRISK 2002 cohort consists of individuals that were randomly drawn from six different geographical regions across Finland and were aged between 25 and 74 years [18]. Microbiome sequencing was performed on faecal samples provided by 7048 participants. From these participants, we excluded those with antibiotic use within 1 month before baseline (*n* = 242), self-reported pregnancy (*n* = 40), a metagenomics read count <50 000 reads (*n* = 13), missing covariate data (*n* = 386) and prevalent ARD (*n* = 125), for a final study sample of 6242 individuals who were included in the analyses. Incident ARD (composite outcome) was defined as developing at least one of the following ARDs: RA, AS or other SCTD. Other SCTD included endpoints, such as vasculitis, lupus, PM, SSc and Sjögren’s disease. The International Classification of Diseases (ICD) codes used to define ARDs are listed in Supplementary Table S1.

The associations of incident ARD with (1) alpha diversity (Shannon index), (2) prevalent taxa (centered log-ratio [CLR]-transformed counts) and (3) prevalent predicted pathways (dichotomized or inverse-rank normalized counts) were assessed using multivariable-adjusted Cox proportional hazard models. Beta diversity, that is dissimilarity of samples in terms of community composition, was estimated with Bray–Curtis index at the species level. Cross-sectional associations between community composition and host variables were quantified with distance-based redundancy analysis (dbRDA), which is a supervised ordination method. The prospective link between microbial community composition and incident ARD was explored using multivariate random survival forest [19], as previously described [20]. Statistical models were adjusted for (1) age and sex and (2) age, sex, BMI, smoking, alcohol use, physical activity, prevalent diabetes, prevalent cardiovascular disease and previous cancer diagnosis. As a sensitivity analysis, we used multivariable-adjusted Cox models to assess the associations of prevalent species with incident ARD for only 10 years of follow-up. *P*-values were corrected for multiple testing using false discovery rate (FDR; Benjamini–Hochberg correction). FDR values < 0.05 were considered statistically significant.

The FINRISK 2002 study complies with the Declaration of Helsinki. The Coordinating Ethics Committee of the Helsinki and Uusimaa University Hospital District approved the FINRISK 2002 study (558/E3/2001). All participants provided written informed consent.

## Results

The characteristics of the study sample are summarized in Table 1. The mean age of the study sample was 49 ± 12.8 years and 46.8% were men. A total of 114 312 person-years of follow-up were included in this study with a median follow-up of 19.8 years. We identified 103 (1.7%) incident RA cases, 16 (0.3%) incident AS cases and 145 (2.3%) incident SCTD cases, for a total of 264 (4.2%) incident ARD cases. The incidence rates were 0.90 per 1000 person‑years for RA, 0.14 for AS, 1.27 for SCTD and 2.31 overall. An overview of the main previous studies examining gut microbiome–ARD associations in humans is provided in Supplementary Table S2.

**Table 1:** Characteristics of the study sample.

Characteristic	All (*N* = 6242)	Incident ARD (*N* = 264)	No incident ARD (*N* = 5978)
Men, *N* (%)	2924 (46.8%)	85 (32.2%)	2839 (47.5%)
Age (years), mean (S.D.)	49 (12.8)	52.7 (12.4)	48.8 (12.8)
BMI (kg/m^2^), mean (S.D.)	26.9 (4.63)	27.2 (4.53)	26.9 (4.64)
Physical activity level, *N* (%)			
Sedentary	1284 (20.6%)	52 (19.7%)	1232 (20.6%)
Light	3499 (56.1%)	153 (58%)	3346 (56%)
Moderate to high	1459 (23.4%)	59 (22.3%)	1400 (23.4%)
Smoking, *N* (%)	1499 (24%)	62 (23.5%)	1437 (24%)
Alcohol intake (g/week), mean (S.D.)	82.5 (124)	66.5 (118)	83.2 (124)
Diabetes, *N* (%)	261 (4.2%)	18 (6.8%)	243 (4.1%)
Cardiovascular disease, *N* (%)	215 (3.4%)	9 (3.4%)	206 (3.4%)
Cancer, *N* (%)	176 (2.8%)	10 (3.8%)	166 (2.8%)
Follow-up time (years), median (minimum, maximum)	19.8 (0.03, 20)	10.2 (0.03, 19.9)	19.8 (0.03, 20)

ARD, autoimmune rheumatic disease.

**Figure 1:**
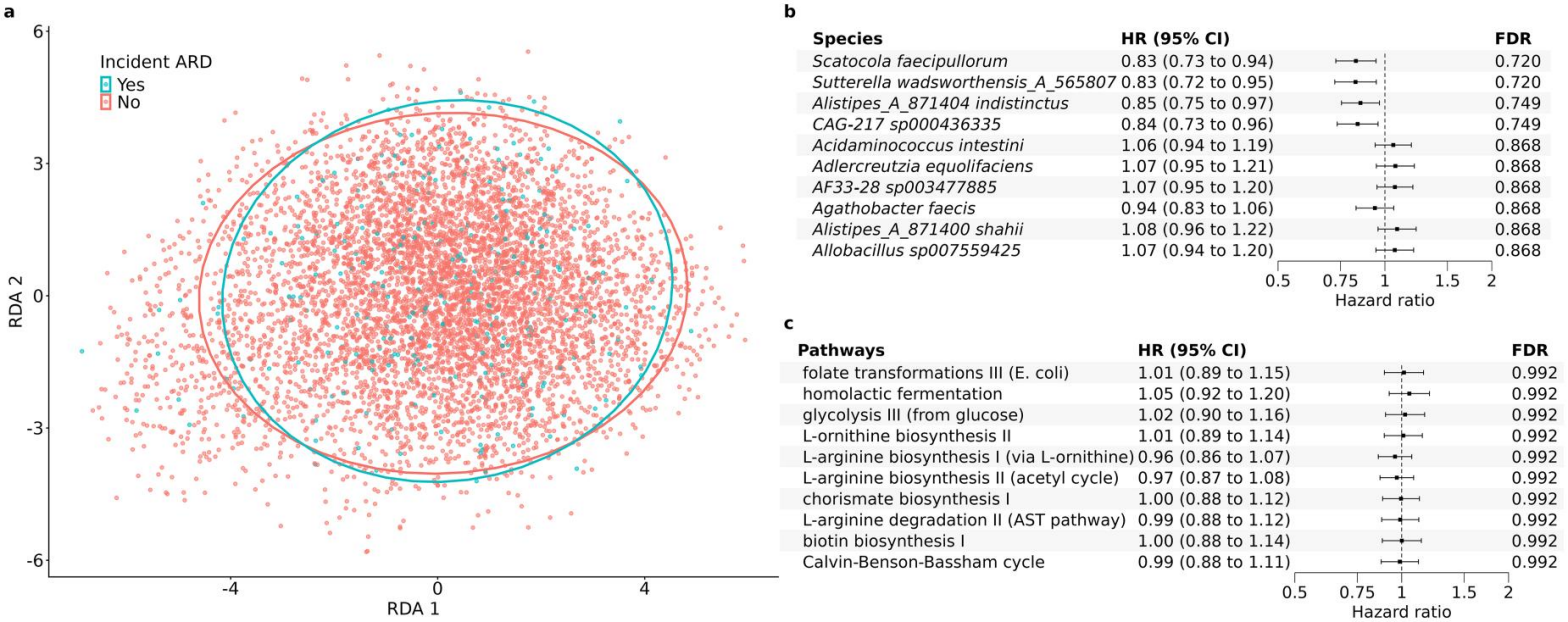
The association between gut microbiome and incident ARD. (**a**) The association between beta diversity (RDA axes 1 and 2) and incident ARD. (**b**) Top 10 species associated with incident ARD and identified using multivariable-adjusted Cox models. (**c**) Top 10 predicted pathways associated with incident ARD and identified using multivariable-adjusted Cox models with dichotomized count data. Hazard ratios are reported per one S.D. change in abundance and are adjusted for age, sex, BMI, smoking, alcohol use, physical activity, prevalent diabetes, prevalent cardiovascular disease and previous cancer diagnosis. For pathway analysis, total read count was added as a covariate to account for its potential confounding role in dichotomous data. ARD, autoimmune rheumatic diseases; RDA, redundancy analysis; HR, hazard ratio; FDR, false discovery rate-adjusted *P*-value

The gut microbiome alpha diversity (Shannon index) was not significantly associated with incident ARD in the age- and sex-adjusted model (hazard ratio [HR] per 1-S.D. change 0.92; 95% CI 0.82–1.03; *P* = 0.13), or in the multivariable-adjusted model (HR per 1-S.D. change 0.92; CI 0.82–1.03; *P* = 0.15). Similarly, no significant association was observed between beta diversity and incident ARD in the age- and sex-adjusted (*R*^2^ = 0.02%; *P* = 0.38) or multivariable-adjusted (*R*^2^ = 0.02%; *P* = 0.39) dbRDA models (Fig. 1a).

We did not observe evidence for a statistically significant association between CLR-transformed abundances of the prevalent taxa (species- and genus-level) and incident ARD (composite outcome) (all FDR > 0.05) (Fig. 1b, Supplementary Tables S3–S6). The top species in the multivariable-adjusted models were *Scatocola faecipullorum* (HR per 1-S.D. change 0.83; CI 0.73–0.94; FDR = 0.72), *Sutterella wadsworthensis_A_565807* (HR per 1-S.D. change 0.83; CI 0.72–0.95; FDR = 0.72), *Alistipes_A_871404 indistinctus* (HR per 1-S.D. change 0.85; CI 0.75–0.97; FDR = 0.75) and *CAG-217 sp000436335* (HR per 1-S.D. change 0.84; CI 0.73–0.96; FDR = 0.75) (Fig. 1b, Supplementary Table S3). These associations had *P*-values below 0.05, however, none reached statistical significance after FDR correction. The associations of the prevalent species with incident inflammatory arthritis (defined as developing either RA or AS) and incident SCTD are presented in Supplementary Tables S7 and S8, respectively. In general, the hazard ratios for the top species identified in the main model were consistent with those observed when incident inflammatory arthritis (Supplementary Table S7) or incident SCTD (Supplementary Table S8) were used as outcome variables.

Of note, we did not find evidence that *Prevotella*, a frequently enriched genus in RA, was associated with incident ARD (*P*-value = 0.5 in the uncorrected analysis for multivariable-adjusted Cox model) (Supplementary Table S5). The estimates of the covariates for one model (top taxon) are shown in Supplementary Table S9. In general, the direction of the effect estimates for the prevalent species remained consistent when the follow-up time was limited to 10 years only (Supplementary Table S10). Moreover, the addition of the prevalent species on top of standard covariates in the random survival forests model did not significantly improve the assessment of incident ARD risk. The *c*-statistic for covariates alone was 0.572 while the *c*-statistic for the combined model was 0.577. The *c*-statistic for prevalent species alone was 0.541. The importance scores of the species are presented in Supplementary Table S11.

No significant associations were detected between predicted MetaCyc pathways and incident ARD using multivariable-adjusted Cox models with dichotomized count data (present vs absent) (Fig. 1c, Supplementary Table S12) or inverse-rank normalized count data (Supplementary Table S13).

## Discussion

In this study, we investigated the potential role of the gut microbiome as a risk factor for incident ARDs in a large population-based cohort. Incident ARD was observed in 264 individuals over a median follow-up period of ∼20 years. The study had adequate statistical power. Using a univariate logistic regression model, we had 80% statistical power to detect an odds ratio of 0.85 or 1.17 for a 1-S.D. change in alpha diversity. We assessed the associations of microbial alpha diversity (Shannon index), community composition, differentially abundant taxa and predicted pathways with the risk of incident ARD. Statistical models were adjusted for covariates known to be associated with ARDs. We did not find evidence that baseline gut microbiome profile is predictive of the risk of developing ARDs (composite outcome) in the general adult population. It is important to note that this study is observational and causal inference cannot be made. Moreover, due to the several limitations, including the use of a composite outcome (rather than individual ARDs) and a single measurement of the microbiome, the null findings of this study should be interpreted with caution.

Previous studies that investigated the relationship between the gut microbiome and ARDs in humans have been mainly cross-sectional and case-control studies [4, 5, 7–12]. Such studies are numerous and provide solid evidence on the link between the gut microbiome and ARDs. Yet, they mainly compare the microbiome diversity and composition between ARD patients and healthy controls, and therefore, are unable to determine whether gut microbiome dysbiosis preceded disease development. Although no single unifying microbial signature for all ARDs can be concluded from these studies, several common gut dysbiosis patterns are observed among multiple ARDs (Supplementary Table S2). These include reduced microbial diversity, increased abundance of Proteobacteria and the loss of short‑chain fatty acids (SCFA)-producing bacteria. Additionally, an expansion of *Prevotella* is frequently reported in RA and AS patients. In our study, however, we did not find evidence of a statistically significant association between the baseline abundance of *Prevotella* and the risk of developing ARD in the adult population.

Several studies have explored whether gut microbiome alterations arise before the onset of ARDs, including RA. One large study reported a link between the genetic risk for RA and the gut microbiome in 1650 participants without the disease [16]. In this cross-sectional analysis that included mainly women, the authors determined the genetic risk of RA using polygenic risk scoring and observed that gut microbiome dysbiosis likely occurs before the development of the disease. Other studies have also focused on gut microbiome changes prior to the onset of RA but were limited to cohorts of high-risk RA individuals [3, 6, 17]. In one particular work, Luo *et al.* compared the gut microbiome of 38 healthy controls and 53 high-risk RA individuals (12 of whom developed RA over a follow-up of 5 years), and reported alterations of the gut microbiome (including an increased abundance of *Eubacterium_brachy_group* and a reduction in the abundance of *Ruminiclostridium_5* and *Ruminococcus_2*) in the preclinical stages of RA. Interestingly, in another study that involved first-degree relatives of RA patients, the authors reported no significant differences in the gut microbiome composition across different preclinical stages of RA [17]. When analyses were limited to subgroups with the most pronounced phenotypes, however, the authors observed modest associations (higher prevalence of Prevotellaceae) in advanced preclinical stages of RA.

One important limitation of our study was the heterogeneity of the outcome variable. We defined the outcome variable as developing at least one of following ARDs: RA, AS or other SCTD. The use of an individual disease as an outcome variable would lead to a more accurate description of the association between the gut microbiome and the outcome variable. However, such approach was not possible in our study due to the low incidence of individual ARDs in the general population. In this case, the use of a single disease as an outcome variable would result in a reduced statistical power, and hence, the inability to draw any meaningful conclusions. Our study highlights the need for larger population-based cohorts that investigate the prospective link between the gut microbiome and individual ARDs.

Other limitations include the inability to generalize the results to other populations since the FINRISK cohort consists of participants with Finnish or European ancestry. Also, we did not adjust for the potential confounding effects of immunomodulatory medications, and although participants with recent antibiotic use were excluded, incomplete recovery of the gut microbiome is still possible. Moreover, in our study, stool samples were collected at one time point only and hence it was not possible to track changes in the gut microbiome composition over the follow-up period. This limitation, combined with the use of a composite outcome, may have masked associations that exist for individual ARDs and therefore, the null results of this study should be interpreted with caution. On the other hand, our strengths include the use of shotgun metagenome sequencing and access to a large randomly selected population sample. In addition, to our knowledge, this was the first study to examine the association between the gut microbiome and the risk of ARDs in a population-based cohort.

## Conclusion

The prior research, mainly based on cross-sectional or case-control studies, has linked gut microbiome dysbiosis to ARDs in patients or individuals at a high risk for the disease. In this study, we found no evidence that baseline gut microbiome composition is prospectively linked with incident ARDs in the general adult population in Finland. It is important to mention that our null results should be interpreted with caution, as they refer to a composite ARD outcome (defined as developing RA, AS or other SCTD) and not to individual ARDs. The low incidence of individual ARDs highlights the need for further research to better understand the relationship between the gut microbiome and individual ARDs in the general adult population.

## Supplementary Material

keag371_Supplementary_Data

## Data Availability

The FINRISK data are available through submitting a request to THL biobank (https://thl.fi/en/research-and-development/thl-biobank/for-researchers/application-process). The underlying code for this study is publicly available and can be accessed via this link https://zenodo.org/records/18429887.
