# Supplementary material for: Prospective analysis on the gut microbiome and the risk of autoimmune rheumatic diseases in the population-based FINRISK 2002 cohort
Source: Rheumatology (Oxford). 2026 Jul 16;65(7):keag371. doi: 10.1093/rheumatology/keag371 (PMC13420491; doi:10.1093/rheumatology/keag371)
Supplement: keag371_Supplementary_Data [file keag371_supplementary_data.zip › rhe-26-0655-File002.docx]

**SUPPLEMENTARY MATERIAL**

**Prospective analysis on the gut microbiome and the risk of autoimmune rheumatic diseases in the population-based FINRISK 2002 cohort**

Hassan Diab^1^, Li-Fang Yeo^1^, Veikko Salomaa^1,2^, Aki Havulinna^3,4^, Leo Lahti^4^, Katariina Pärnänen^4,5^, Rob Knight^6,7,8,9,10,11^, Joonatan Palmu^1,2,12^, Teemu Niiranen^1,2,13^

^1^ Department of Internal Medicine, University of Turku, Turku, Finland.

^2^ Department of Public Health, Finnish Institute for Health and Welfare, Helsinki, Finland.

^3^ Faculty of Medicine, Research Programs Unit, Clinical and Molecular Metabolism (CAMM), University of Helsinki, Helsinki, Finland.

^4^ Department of Computing, University of Turku, Turku, Finland.

^5^ Department of Microbiology, University of Helsinki, Helsinki, Finland.

^6^ Department of Pediatrics, University of California San Diego, San Diego, CA, USA.

^7^ Center for Microbiome Innovation, Joan and Irwin Jacobs School of Engineering, University of California San Diego, La Jolla, CA, USA.

^8^ Department of Bioengineering, University of California San Diego, La Jolla, CA, USA.

^9^ Department of Computer Science and Engineering, University of California San Diego, La Jolla, CA, USA.

^10^ Halıcıoğlu Data Science Institute, University of California San Diego, La Jolla, CA, USA.

^11^ Hong Kong University of Science and Technology Jockey Club Institute for Advanced Study, Hong Kong University of Science and Technology, Hong Kong SAR, China.

^12^ Heart Center, Turku University Hospital, Turku, Finland.

^13^ Division of Medicine, Turku University Hospital, Turku, Finland.

Corresponding author: Hassan Diab, Department of Internal Medicine, University of Turku, Turku, Finland; Email: [hassan.h.diab@utu.fi](mailto:hassan.h.diab@utu.fi)

**Methods**

*Study sample*

The FINRISK 2002 cohort is part of the national FINRISK surveys that were conducted every five years between 1972 and 2012, and were aimed at studying the risk factors of non-communicable diseases in the Finnish population.^1^ The FINRISK 2002 cohort consists of individuals that were randomly drawn from six different geographical regions across Finland and were aged between 25 and 74 years. In 2002, a random sample of 13,437 individuals was selected from the national population register, of whom 8,799 completed the health examination. Microbiome sequencing was done for a total of 7,048 participants who provided fecal samples. From these participants, we excluded those with antibiotic use within one month before baseline (n=242), self-reported pregnancy (n=40), a metagenomics read count <50,000 reads (n=13), missing covariate data (n=386), and prevalent ARD (n=125), for a final study sample of 6,242 individuals who were included in the analyses.

*Questionnaire and health examination*

Participants responded to a detailed questionnaire that contained questions on family history, use of medications, diet, diagnoses, functional capacity, and other health behaviors. Health examination was performed to participants at local study centers and included anthropometric measurements and blood sample collection. At the end of the health examination, participants were instructed on how to provide a stool sample at home.

*Microbiome sequencing from stool samples*

Stool samples were collected by participants at home and then mailed, overnight, to the Finnish Institute for Health and Welfare where they were stored at -20°C. In 2017, the samples underwent metagenomic sequencing at the University of California San Diego. The microbiome analysis was performed by mapping the untargeted shallow shotgun metagenomic reads against reference databases, as previously described.^2^ Greengenes2 database^3^ was used for taxonomy assignment and the data was imported into an R TreeSummarizedExperiment container^4^ for downstream analysis. MetaCyc^5^ database in the HUMAnN3 pipeline (HUMAnN v3.0.0 with the ChocoPhlAn mpa_v30_CHOCOPhlAn_201901 and UniRef90 databases)^6^ was used to annotate functional pathway abundances.

*Outcome variable*

The incidence of ARD was used as an outcome variable. Incident ARD was defined as developing at least one of following ARDs: RA, AS, or other SCTD. Other SCTD included endpoints, such as vasculitis, polymyositis, systemic sclerosis, and Sjögren’s disease. The International Classification of Diseases (ICD) codes used to define ARDs are listed in **Supplementary Table 1.**

*Covariates*

Body mass index (BMI, kg/m^2^) was calculated using height and weight values that were measured by centrally trained nurses. Smoking was self-reported and defined as current daily smoking. Self-reported alcohol consumption was calculated as the average weekly pure alcohol use in grams during the past 12 months. For physical activity, participants answered a 4‐option multiple choice question that included the following options for leisure‐time activity: (1) sedentary, (2) light activity for >4 hours per week, (3) fitness training or other strenuous exercise for >3 hours per week, and (4) competitive sports.^7^ Categories three and four were combined due to the small number of individuals in category four. The International Classification of Diseases (ICD) codes used to define diabetes, cardiovascular disease, and cancer are available in **Supplementary Table 1.**

*Statistical methods*

The microbiome data was analyzed using mia R/Bioconductor package.^8^ Alpha diversity was defined with Shannon index at species-level. We tested differences in community composition (beta diversity) between the groups with distance-based redundancy analysis (dbRDA) based on Bray-Curtis dissimilarity calculated at the species level. For this analysis, the species-level relative abundance table was used as an outcome (dependent) variable while incident ARD was used as a predictor (independent variable). Differential abundance analysis was performed for each prevalent taxon (172 genera and 259 species) which were defined as being prevalent in at least 5% of the sample population with a relative abundance over 0.1%. Centered log-ratio (CLR) transformation was applied to the abundances of the prevalent taxa prior to the differential abundance analysis. The associations of incident ARD with alpha diversity and prevalent taxa (CLR-transformed counts) were assessed using multivariable-adjusted Cox proportional hazard models. We used GPower^9^ to calculate the statistical power for the univariate logistic regression model assessing the association between alpha diversity and incident ARD. As a sensitivity analysis, we used multivariable-adjusted Cox models to assess the associations of the prevalent species with incident ARD for only 10 years of follow-up. We also assessed the relationship between prevalent species and 1) incident inflammatory arthritis (defined as developing either RA or AS) and 2) incident SCTD, using multivariable-adjusted Cox models.

The link between microbial community composition and incident ARD was also explored using multivariate Random Survival Forest^10^ (R package randomForestSRC) with CLR-transformed counts of the prevalent species, as previously described.^2^ The performance of the model was assessed using Harrell’s *c*-statistic.^11^ The predictor sets included microbiome (prevalent species), covariates, and microbiome plus covariates (combined model). Prediction errors were estimated using out-of-bag estimators.

For functional analyses, we used general predicted MetaCyc pathways^5^ that were prevalent in at least 5% in the sample population (with a detection threshold of zero). We excluded superpathways, species-associated pathways, and non-bacterial pathways from the analysis. The pathway counts were 1) dichotomized or 2) inverse-rank normalized to account for the highly sparse and zero-enriched nature of the pathway data. For dichotomous transformation, counts that were greater than zero were transformed to one. Total read count was added as a covariate to account for its potential confounding role in dichotomous data.

All models were adjusted for age, sex, BMI, smoking, alcohol use, physical activity, prevalent diabetes, prevalent cardiovascular disease, and previous cancer diagnosis. Analyses were also performed with age- and sex-adjusted models. P-values were corrected for multiple testing using False Discovery Rate (FDR; Benjamini–Hochberg correction). FDR values < 0.05 were considered statistically significant. R version 4.5.2 was used for all statistical analyses.

**References**

1. Borodulin, K. *et al.* Cohort Profile: The National FINRISK Study. *Int. J. Epidemiol.* **47**, 696–696i (2018).

2. Salosensaari, A. *et al.* Taxonomic signatures of cause-specific mortality risk in human gut microbiome. *Nat. Commun.* **12**, 2671 (2021).

3. McDonald, D. *et al.* Greengenes2 unifies microbial data in a single reference tree. *Nat. Biotechnol.* **42**, 715–718 (2024).

4. Huang, R. *et al.* TreeSummarizedExperiment: a S4 class for data with hierarchical structure [version 2; peer review: 3 approved]. *F1000Res.* **9**, 2–43 (2021).

5. Caspi, R. *et al.* The MetaCyc database of metabolic pathways and enzymes - a 2019 update. *Nucleic Acids Res.* **48**, D455–D453 (2020).

6. Beghini, F. *et al.* Integrating taxonomic, functional, and strain-level profiling of diverse microbial communities with bioBakery 3. *Elife* **10**, e65088 (2021).

7. Kullberg, R. F. J. *et al.* Association between butyrate-producing gut bacteria and the risk of infectious disease hospitalisation: results from two observational, population-based microbiome studies. *Lancet Microbe* **5**, 100864 (2024).

8. Borman, T. *et al.* Orchestrating Microbiome Analysis with Bioconductor. *bioRxiv* 2025.10.29.685036 (2025) doi:10.1101/2025.10.29.685036.

9. Erdfelder, E., FAul, F., Buchner, A. & Lang, A. G. Statistical power analyses using G*Power 3.1: Tests for correlation and regression analyses. *Behav. Res. Methods* **41**, 1149–1160 (2009).

10. Ishwaran, H., Kogalur, U. B., Blackstone, E. H. & Lauer, M. S. Random survival forests. *Ann. Appl. Stat.* **2**, 841–860 (2008).

11. Harrell, F. E., Califf, R. M., Pryor, D. B., Lee, K. L. & Rosati, R. A. Evaluating the Yield of Medical Tests. *JAMA* **247**, 2543–2546 (1982).
